# The Role of Scavenger Receptor BI in Sepsis

**DOI:** 10.3390/ijms252413441

**Published:** 2024-12-15

**Authors:** Dan Hao, Jian-Yao Xue, Qian Wang, Ling Guo, Xiang-An Li

**Affiliations:** 1 Department of Pharmacology and Nutritional Sciences, University of Kentucky College of Medicine, Lexington, KY 40536, USA; 2 Saha Cardiovascular Research Center, University of Kentucky, Lexington, KY 40536, USA; 3 Lexington VA Healthcare System, Lexington, KY 40502, USA; 4 Department of Physiology, University of Kentucky, Lexington, KY 40536, USA

**Keywords:** scavenger receptor BI, SR-BI, HDL, sepsis, adrenal stress response, glucocorticoid, precision medicine

## Abstract

Sepsis is a life-threatening condition resulting from a dysregulated host response to infection. Currently, there is no effective therapy for sepsis due to an incomplete understanding of its pathogenesis. Scavenger receptor BI (SR-BI) is a high-density lipoprotein (HDL) receptor that plays a key role in HDL metabolism by modulating the selective uptake of cholesteryl ester from HDL. Recent studies, including those from our laboratory, indicate that SR-BI protects against sepsis through multiple mechanisms: (1) preventing nitric oxide-induced cytotoxicity; (2) promoting hepatic lipopolysaccharide (LPS) clearance and regulating cholesterol metabolism in the liver; (3) inhibiting LPS-induced inflammatory signaling in macrophages; and (4) mediating the uptake of cholesterol from HDL for inducible glucocorticoid (iGC) synthesis in the adrenal gland, which controls systemic inflammatory response. In this article, we review the roles of SR-BI in sepsis.

## 1. Introduction

**Figure 1:**
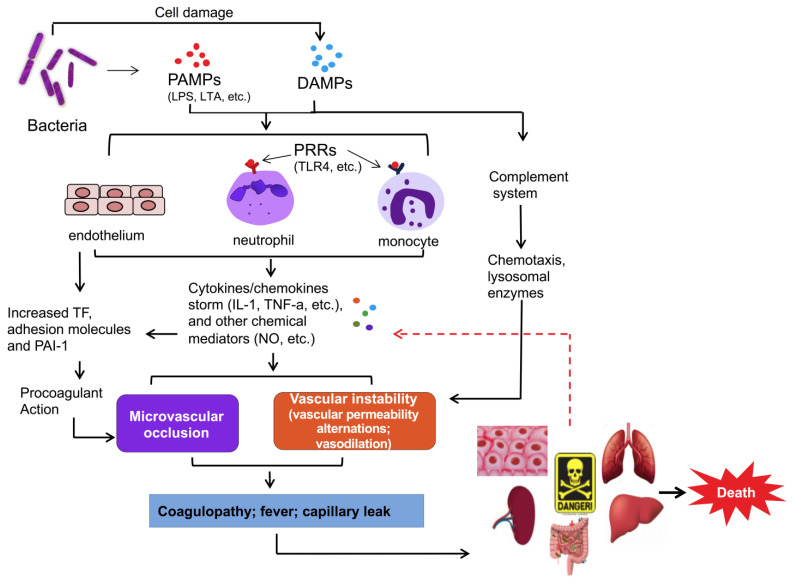
The pathogenesis of sepsis. Upon infection, pattern recognition receptors (PRRs) on immune cells recognize and bind to specific microbial structures known as PAMPs or DAMPs. The activation of the innate immune system results in the release of cytokines/chemokines, nitric oxide, and oxygen radicals, and complement system activation. This process leads to coagulation due to endothelial damage and the rapid release of pro-inflammatory cytokines. The complement system and coagulation cascades subsequently reduce vascular stability and cause microvascular blockages, resulting in coagulation, fever, capillary leakage, and ultimately multiple-organ failure. These factors drive the typical inflammatory response underlying sepsis’ pathophysiology. LPS, lipopolysaccharide; LTA, lipoteichoic acid; PAMPs, pathogen-associated molecular patterns; DAMPs, damage-associated molecular patterns; TLR4, toll-like receptor 4; TF, tissue factor; PAI-1, plasminogen activator inhibitor-1; IL-1, interleukin-1; TNF-1α, tumor necrosis factor-1 alpha; NO, nitric oxide.

Sepsis is a major health issue, affecting 49 million people annually [1]. Sepsis results from a dysregulated host response to infection [2]. Upon infection (Figure 1), pattern recognition receptors (PRRs) on immune cells recognize and bind to the molecular structures of the invading microorganism, known as pathogen- or damage-associated molecular patterns (PAMPs and DAMPs). These trigger the activation of the innate immune system, leading to the release of cytokines, chemokines, nitric oxide, and oxygen radicals, and complement system activation. Concurrently, coagulation is triggered by endothelial damage and amplified by pro-inflammatory cytokines. The complement system and coagulation cascades cause vascular instability, microvascular occlusion, coagulation, fever, and capillary leakage, ultimately contributing to the development of multiple-organ failure [3,4,5]. Despite extensive efforts, hundreds of sepsis therapies have proven to be unsuccessful. An incomplete understanding of the pathogenesis of sepsis may contribute to these failed efforts.

Scavenger receptor BI (SR-BI) is a 75 kDa cell surface glycoprotein that is highly expressed in various tissues, including the liver, endothelial cells, macrophages, and steroidogenic tissues [6,7,8]. It was initially identified by Dr. Krieger’s group as a receptor that binds to acetylated low-density lipoprotein (LDL), oxidized LDL, and native LDL in vivo [9]. Two years later, their research revealed that SR-BI also binds to high-density lipoprotein (HDL) and facilitates the selective uptake of cholesteryl esters from HDL particles into cells. This remarkable function led to its designation as “the HDL receptor” [10,11,12,13,14]. SR-BI-mediated uptake of cholesteryl ester from HDL is essential for reverse cholesterol transport in the liver, where HDL collects cholesterol from peripheral tissues and transfers it to hepatic SR-BI for disposal in bile. Mice deficient in SR-BI have elevated HDL levels, female infertility, and autoimmune disorders with aging [15], and are susceptible to atherosclerosis [16,17,18,19,20]. Similarly, humans with loss-of-function mutations in SR-BI exhibit impaired uptake of cholesteryl esters from HDL, elevated HDL levels, and an increased risk of coronary heart disease [21,22]. This indicates that SR-BI has similar functions in both humans and rodents (please refer to SR-BI reviews [23,24,25]).

Dr. Li’s laboratory first reported the protective effect of SR-BI against sepsis. They found that SR-BI-null mice have significantly higher mortality in LPS-induced sepsis (90% mortality in SR-BI-null mice versus 0% in wild-type mice) [26]. Using a cecal ligation and puncture (CLP)-induced sepsis model, they confirmed the protective role of SR-BI in sepsis [27]. Subsequent research from a number of laboratories has elucidated various mechanisms through which SR-BI exerts its protective functions, which will be explored in the following discussion.

## 2. Scavenger Receptor BI Protects Against Nitric-Oxide-Induced Cytotoxicity

It has been shown that HDL can enhance endothelial function and nitric oxide (NO)-dependent relaxation in aortas. However, this beneficial effect is not observed in the aortas of SR-BI-null mice, emphasizing the crucial role of SR-BI in this process. Further investigation has shown that HDL activates eNOS through SR-BI [28,29,30,31]. NO is a highly reactive molecule produced by nitric oxide synthase (NOS). It defends against pathogens but can become cytotoxic in excess [32]. It reacts with superoxide to form the hazardous peroxynitrite, leading to nitrotyrosine formation and cytotoxicity [33]. This process, called nitric oxidative stress, underlies diseases like septic shock [34], atherosclerosis [35], Alzheimer’s disease [36], and diabetes [37]. Using Chinese Hamster Ovary (CHO) cell lines expressing wild-type or mutant SR-BI, Dr. Li’s laboratory demonstrated SR-BI’s protection against NO-induced cytotoxicity [26]. Using SR-BI-null mice, they found that SR-BI significantly reduces protein tyrosine nitration in the aorta and liver. To further investigate the physiological and pathological significance of SR-BI’s protection against NO-induced cytotoxicity, they used a lipopolysaccharide (LPS) challenge as a model. When challenged with a low dose of LPS, 90% of SR-BI-knockout mice died, whereas none of the wild-type littermates died. An LPS challenge induces endotoxemia and serves as a model of sepsis. These findings establish SR-BI’s protective role against sepsis. 

## 3. Scavenger Receptor BI Mediates Lipopolysaccharide Clearance

SR-BI also functions as a receptor for LPS, a key factor in septic shock, and it is highly expressed in the liver, the primary site for LPS clearance. It is therefore considered pivotal in facilitating LPS clearance by the liver. Evidence shows that SR-BI binds and internalizes LPS in HeLa cells and macrophages, facilitating LPS clearance into hepatocytes and reducing the LPS burden [38]. Guo et al. demonstrated the role of SR-BI in LPS recruitment and clearance in CLP-induced sepsis [27]. Cai et al. found that SR-BI-null mice exhibited reduced plasma LPS clearance into the liver and hepatocytes [39], further emphasizing the crucial function of SR-BI in facilitating LPS clearance. Hepatic SR-BI is crucial for LPS clearance during sepsis, as observed in mice with hepatic SR-BI deficiency (Scarb1^I179N^ mice), experiencing a three-fold increase in serum LPS levels during CLP-induced sepsis compared to control mice [40]. These findings highlight the significant role of SR-BI in promoting LPS clearance during sepsis.

## 4. Scavenger Receptor BI-Mediated Cholesterol Metabolism Is Required for Protection Against Sepsis

Lipid metabolism often goes haywire in septic patients, leading to complications. Increased lipolysis releases free fatty acids (FFAs) into the bloodstream, but their utilization is impaired due to mitochondrial dysfunction and reduced enzyme activity. The systemic inflammatory response inhibits lipoprotein lipase (LPL), altering lipoprotein profiles and exacerbating inflammation. Oxidative stress further contributes to lipid peroxidation, damaging cell membranes and worsening lipid metabolism. These disruptions contribute to the pathophysiology of sepsis and affect patient outcomes [41,42]. 

Reverse cholesterol transport (RCT) plays a vital role in managing cholesterol levels in the bloodstream. During RCT, HDL gathers cholesterol from peripheral tissues and delivers it to the liver’s SR-BI receptors. SR-BI then facilitates the uptake of cholesteryl esters from HDL, which are eventually excreted via bile. In a recent study by Wang et al., researchers used liver-specific SR-BI-null (AlbCreSR-BI^fl/fl^) mice, which have impaired RCT, to explore how this process affects sepsis [43]. They discovered that these mice were much more vulnerable to CLP-induced polymicrobial sepsis, with a survival rate of only 14.3% compared to 80% in their SR-BI^fl/fl^ littermates. Mechanistically, sepsis disrupted cholesterol metabolism in these mice, leading to a 4.8-fold increase in free cholesterol (FC) levels and a 4-fold increase in the FC/cholesteryl ester (CE) ratio compared to the control group. This disruption resulted in hemolysis and death. Interestingly, when the cholesterol-lowering drug probucol was administered, it normalized FC levels and the FC/CE ratio, significantly improving survival rates in the CLP-AlbCreSR-BI^fl/fl^ mice. However, probucol treatment had the opposite effect in CLP-LDLR-knockout mice, which had elevated CE levels and a low FC/CE ratio, reducing their survival. These findings underscore that elevated FC levels and a high FC/CE ratio are significant risk factors for sepsis. Therefore, targeting these elevated FC levels and the FC/CE ratio could be a promising therapeutic strategy for managing sepsis.

## 5. Scavenger Receptor BI Regulates Inflammatory Response

Macrophages are a major cell type responsible for cytokine production, and SR-BI appears to regulate their inflammatory signaling. The expression of SR-BI suppresses inflammatory cytokine production in LPS-stimulated macrophages by modulating the TLR4-NF-κB pathway. Further study demonstrated that the regulation of SR-BI involves JNK and P38 signaling pathways [44]. Hematopoietic SR-BI deficiency leads to increased cytokine production in response to LPS, indicating its role in controlling in vivo inflammation. The inhibitory effects of SR-BI on the macrophage inflammation protect against septic death, exemplified by SR-BIC323G overexpression (a mutant SR-BI deficient in glucocorticoid generation but capable of suppressing TLR4-NF-κB signaling) significantly improving survival in SR-BI-deficient mice during a CLP challenge [27]. A recent bulk RNA-seq analysis found that adrenal SR-BI-knockout mice displayed hyperinflammatory responses during sepsis due to the transcriptional dysregulation of AP-1 and NK-κB. GC therapy effectively rescued these mice [45]. These findings underscore SR-BI’s role in inflammation and its vital role in moderating inflammation for protection against sepsis-induced death. 

Using the LPS model, Cai et al. confirmed that SR-BI-knockout mice are susceptible to LPS-induced endotoxic death, which is associated with a systemic hyperinflammatory response [39]. They further found that a deficiency in SR-BI leads to low levels of glucocorticoid (GC) production, which contributes to the uncontrolled inflammatory response (discussed further below).

## 6. Scavenger Receptor BI Regulates Adrenal Stress Response in Sepsis

In the adrenal gland, glucocorticoid (GC) production is markedly induced in response to septic stress and serves as potent inflammatory modulators during sepsis [46]. We refer to this stress-induced GC (iGC) as the adrenal stress response. GC is derived from intracellular cholesterol, which comes from three resources: (1) uptake from LDL through LDL receptors; (2) uptake from HDL through SR-BI; and (3) de novo synthesis. The SR-BI-HDL pathway appears to play a key role in iGC production. SR-BI-deficient mice maintain normal basal GC levels under physiological conditions, but cannot generate iGC under stress conditions induced by factors like LPS [39], adrenocorticotropic hormone (ACTH) stimulation [39], CLP [27,47,48], or long-term fasting [49,50]. SR-BI-null mice exhibit normal expression in key genes related to cholesterol de novo synthesis [39]. Given that rodents mainly have HDL with very low LDL levels in circulation, we generated humanized SR-BI-/- ApoBtg mice (SR-BI-null mice express with ApoB) with high LDL in circulation. The mice also failed to produce iGC in response to ACTH stimulation or under sepsis conditions [51]. Regarding the role of SR-BI in humans, an early study showed that human carriers’ SR-BI P297S mutant, which has a 50% reduction in the uptake of cholesterol from HDL, displays a 50% reduction in iGC production to ACTH stimulation [22]. In contrast to SR-BI, a 50% reduction in LDL receptors in familial hypercholesterolemia patients does not hinder cholesterol delivery to the adrenal cortex [52]. These studies establish the SR-BI-HDL pathway as a key regulator of iGC production in sepsis. 

## 7. Scavenger Receptor BI-Mediated Adrenal Stress Response Is an Essential Host Response for Protection Against Sepsis

SR-BI-null mice have a normal corticosterone level under physiologic conditions but lack iGC production during sepsis. This makes SR-BI-null mice a useful model to investigate the role of iGC in sepsis. Using whole-body SR-BI-null mice and the LPS endotoxemia model, Cai et al. showed that a lack of iGC production results in significantly elevated cytokine levels in circulation [39]. Using SF1CreHypoSR-BI^fl/fl^ and the CLP sepsis model, Dr. Huby’s group showed that these mice are more susceptible to CLP-induced septic death than control (HypoSR-BI^fl/fl^) mice [47]. Using adrenal transplant-generated adrenal-specific SR-BI-null mice and the CLP sepsis model [48], Dr. Li’s laboratory showed that mice deficient in adrenal SR-BI fail to produce iGC production in response to a CLP challenge and are more susceptible to CLP-induced septic death. Importantly, GC treatment two hours post CLP effectively rescued adrenal-specific SR-BI-null mice, but caused increased mortality in wild-type control mice. Since adrenal transplantation may disrupt catecholamine production by the adrenal gland, Dr. Li’s laboratory generated new adrenal-specific SR-BI-null (SF1CreSR-BI^fl/fl^) mice using floxed SR-BI mice [53,54]. They showed that adrenal SR-BI-specific knockout mice have impaired iGC production in response to ACTH stimulation and to CLP-induced sepsis. They demonstrated that while both wild-type and SF1CreSR-BI^fl/fl^ mice exhibit a hyperinflammatory phenotype in the early stages of sepsis, iGC keeps the inflammatory response under control in wild-type mice. In contrast, SF1CreSR-BI^fl/fl^ mice experience uncontrolled hyperinflammation due to the lack of iGC. These mice are more susceptible to CLP-induced sepsis. Supplementation with a low stress dose of GC in SF1CreSR-BI^fl/fl^ mice controls the inflammatory response and rescues the mice. However, SR-BI^fl/fl^ mice receiving GC treatment exhibited significantly less survival rates compared to SR-BI^fl/fl^ mice without GC treatment. 

Dr. Li’s laboratory further assessed the importance of SR-BI-mediated iGC production in pediatric sepsis using 21-day-old mice [45]. Mice deficient in adrenal SR-BI were susceptible to both CLP and cecal slurry-induced septic death. SF1CreSRBI^fl/fl^ mice featured persistent inflammatory responses, and were effectively rescued by administering GC two hours post CLP. GC treatment did not improve survival in CLP-challenged wild-type mice. Using an RNA-seq analysis, they found that the lack of iGC production in SF1CreSRBI^fl/fl^ mice caused persistent inflammatory responses, primarily due to the transcriptional dysregulation of activator protein 1 (AP-1) and nuclear factor kappa B (NF-κB) [45]. In addition, they found that iGC functions to control cytokine-induced secondary inflammatory response [45].

It is of interest to discuss the effect of pretreatment of SR-BI KO mice with GC in LPS and CLP models. In an LPS endotoxemia model, Cai et al. [39] treated the mice with corticosterone via drinking water, 8 h prior to LPS injection, which effectively improved survival rates and physical condition in SR-BI-null mice. In a CLP-induced septic mice model [27], Guo et al. showed that corticosterone supplementation 8 h before CLP surgery does not rescue SR-BI-null mice from CLP-induced septic death. This may result from differences in the immune response between CLP-induced sepsis models and LPS-induced endotoxemia models. LPS injection simply triggers the release of inflammatory cytokines, which can be controlled by corticosterone supplementation; however, CLP-induced sepsis involves bacterial infection and necessary cytokine production for immune defense. Corticosterone given 8 h before CLP surgery could potentially leave the host vulnerable by suppressing the essential early immune response, making glucocorticoid pretreatment unfeasible for sepsis therapy. In another study, Leelahavanichkul et al. administered a high dose of dexamethasone to SR-BI KO and wild-type control mice 24 h before CLP, and found increased survival in SR-BI KO mice [55]. Unfortunately, the data did not support such a conclusion due to flaws in the experimental design [56]. For example, dexamethasone pretreatment completely eliminated SR-BI expression in the adrenal gland in wild-type control mice; the study used SR-BI-null mice on a C57BL/6/129 mixed background but used C57BL/6N mice as controls. Given that C57BL/6N mice are significantly more susceptible to sepsis than mice with a mixed background and that the adrenal SR-BI plays a key role in protection against sepsis, the increased survival in SR-BI KO mice is more likely due to the effect of GC pretreatment and the effect of the background. These early studies clearly showed that GC treatment to septic wild-type mice, especially pretreatment, is harmful. 

## 8. Conclusions

**Figure 2:**
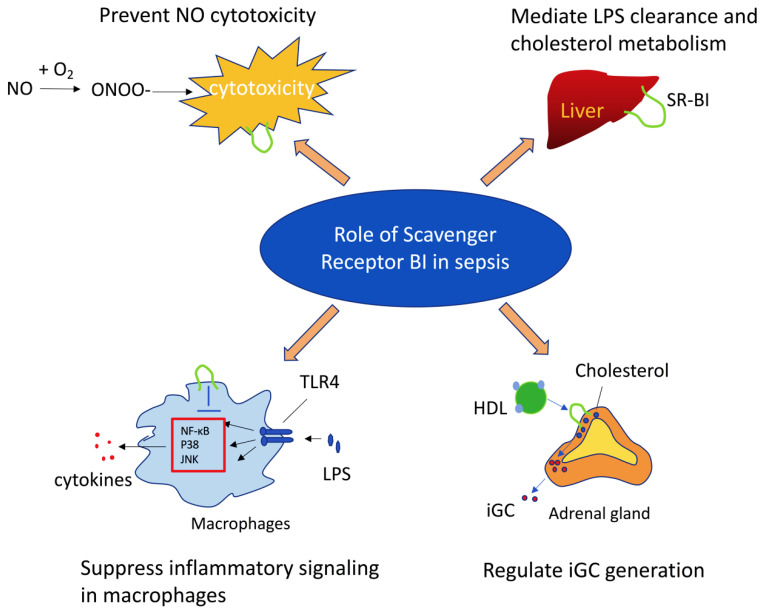
Summary of SR-BI protection against sepsis. SR-BI exerts its protective effect in sepsis through multiple mechanisms: (1) prevent NO-induced cytotoxicity; (2) mediate LPS clearance and cholesterol metabolism in liver; (3) suppress LPS-induced inflammatory signaling in macrophages; (4) mediate uptake of cholesterol from HDL for iGC synthesis in adrenal gland, which controls systemic inflammatory response.

SR-BI protects against sepsis through multiple mechanisms (Figure 2): (1) SR-BI protects against NO-induced cytotoxicity; (2) SR-BI mediates hepatic LPS clearance and cholesterol metabolism; (3) macrophage SR-BI inhibits LPS-induced inflammatory signaling; (4) adrenal SR-BI mediates the uptake of cholesterol from HDL for iGC synthesis, which controls the systemic inflammatory response.

## 9. Future Direction 

Extensive clinical trials have failed to improve survival in septic patients [57]. A limitation is that these therapies were applied non-selectively to all septic patients. However, septic patients have heterogeneous clinical endotypes. Given the complexity of sepsis, emerging voices [58,59,60], including ours [45,48,53,61], have called for a precision medicine approach to sepsis therapy. Targeting a subgroup of patients with a specific endotype is a key component of a successful precision medicine approach [62,63]. 

Dysregulated lipid metabolism is common in septic patients [64], and statins have been tested as a sepsis therapy. However, the efficacy of statins is controversial [65,66,67,68,69]. A potential issue is that statins have been used indiscriminately in all septic patients, without accounting for variations in cholesterol metabolic subtypes. A mechanistic study using hepatic SR-BI-null mice as a model of dysregulated reverse cholesterol transport demonstrated that elevated free cholesterol, with a high free cholesterol/cholesteryl ester ratio, is a risk factor for sepsis [43]. Lowering cholesterol with probucol improved survival in hepatic SR-BI-null mice. Thus, probucol or statin treatment for septic patients with high free cholesterol levels and a high free cholesterol/cholesteryl ester ratio may offer an effective sepsis therapy.

Glucocorticoid (GC) therapy has been frequently used in septic patients, but its effectiveness remains hotly debated [70,71]. Current guidelines suggest GC therapy for septic patients experiencing shock [71]. While GC plays an important role in regulating blood pressure, this recommendation is largely based on clinical observations. Hypotension results from organ dysfunction. Given that hyperinflammation significantly contributes to organ injury, and mechanistic studies using adrenal SR-BI-null mice as an adrenal insufficiency model have demonstrated that GC functions control inflammation, and adrenal insufficiency is a risk factor for sepsis, we advocate for a precision medicine approach to guide GC therapy for sepsis—timely and selectively applying GC therapy to patients with adrenal insufficiency, not without.

## Data Availability

Not applicable.
